# Identifying the areas of low self-reported confidence of internal medicine residents in geriatrics: a descriptive study of findings from a structured geriatrics skills assessment survey

**DOI:** 10.1186/s12909-022-03934-2

**Published:** 2022-12-15

**Authors:** Kristina Marie Kokorelias, Grace Leung, Namirah Jamshed, Anna Grosse, Samir K. Sinha

**Affiliations:** 1 Division of Geriatric Medicine, Department of Medicine, Sinai Health System and University Health Network, Suite 475 - 600 University Avenue, Toronto, Ontario M5G 1X5 Canada; 2 Division of Geriatric Medicine, Department of Medicine, University of Toronto, Medical Sciences Building, 1 King’s College Cir, Toronto, Ontario M5S 1A8 Canada; 3 Division of Geriatric Medicine, UT Southwestern Medical Center, Dallas, TX USA; 4 Division of Geriatric Medicine and Gerontology, Johns Hopkins University School of Medicine, Baltimore, USA

**Keywords:** Geriatrics, Medical education, Training, Needs assessment, Internal medicine, Residency

## Abstract

**Background:**

Currently, no standardized methods exist to assess the geriatric skills and training needs of internal medicine trainees to enable them to become confident in caring for older patients. This study aimed to describe the self-reported confidence and training requirements in core geriatric skills amongst internal medicine residents in Toronto, Ontario using a standardized assessment tool.

**Methods:**

This study used a novel self-rating instrument, known as the Geriatric Skills Assessment Tool (GSAT), among incoming and current internal medicine residents at the University of Toronto, to describe self-reported confidence in performing, teaching and interest in further training with regard to 15 core geriatric skills previously identified by the American Board of Internal Medicine.

**Results:**

190 (75.1%) out of 253 eligible incoming (Year 0) and current internal medicine residents (Years 1–3) completed the GSAT. Year 1–3 internal medicine residents who had completed a geriatric rotation reported being significantly more confident in performing 13/15 (*P* < 0.001 to *P* = 0.04) and in teaching 9/15 GSAT skills (*P* < 0.001 to *P* = 0.04). Overall, the residents surveyed identified their highest confidence in administering the Mini-Mental Status Examination and lowest confidence in assessing fall risk using a gait and balance tool, and in evaluating and managing chronic pain.

**Conclusion:**

A structured needs assessment like the GSAT can be valuable in identifying the geriatric training needs of internal medicine trainees based on their reported levels of self-confidence. Residents in internal medicine could further benefit from completing a mandatory geriatric rotation early in their training, since this may improve their overall confidence in providing care for the mostly older patients they will work with during their residency and beyond.

**Supplementary Information:**

The online version contains supplementary material available at 10.1186/s12909-022-03934-2.

## Background

The rapid growth in the number of persons aged 65 years and older has been well documented, as have concerns of how global healthcare systems will respond to meet the complex medical needs of growing aging populations [1–3]. Older patients often present with unique healthcare considerations that include interacting comorbidities [4], increased vulnerability to the adverse effects of polypharmacy [5, 6], frailty [7] and a poorer prognosis for different conditions in comparison with younger patients [8–10]. While it would be ideal if the number of geriatricians corresponded with specialist-to-aging-population requirements, the current and future predicted number of geriatricians does not align with existing and future needs [11, 12]. As such, there has been growing recognition of the need to ensure that all physicians working with older persons are confident and capable of delivering appropriate geriatric care [13–16]. Given that the majority of care general internists provide is to older adults [17–19], it is crucial that internal medicine residency programs focus on preparing the next generation of physicians to address the specific needs of older patients [20, 21].

With a rapidly aging population, it is reasonable to expect that all internal medicine specialists graduating from residency training programs acquire an appropriate amount of knowledge, skill and exposure to caring for older adults, so they can be confident in providing care for them. Despite this, current literature suggests that internal medicine physicians may not feel comfortable in some areas of geriatric medicine, such as deprescribing guideline-recommended therapies to avoid polypharmacy [22], addressing frailty [16] and managing polymorbidity and complexity [23]. To assist internal medicine residents, a variety of novel geriatric training programs and curriculums have been developed [24–28]. Historically, these programs have often been evaluated through knowledge-based testing or surveys of residents’ experiences [24, 27–29]. Measuring a trainees’ self-confidence in the performance of core geriatric skills is also important, as their level of self-confidence can potentially predict performance success, indicate their satisfaction with their training and impact career choices [30–32]. Whereas validated tools measuring residents’ self-confidence exist for other specialties like surgery [33, 34], there is no such tool for geriatric medicine. In the absence of a robust, standardized method to assess the self-reported confidence of internal medicine trainees in performing or teaching core geriatric skills, it is difficult to ascertain areas for improvement.

**Fig. 1:**
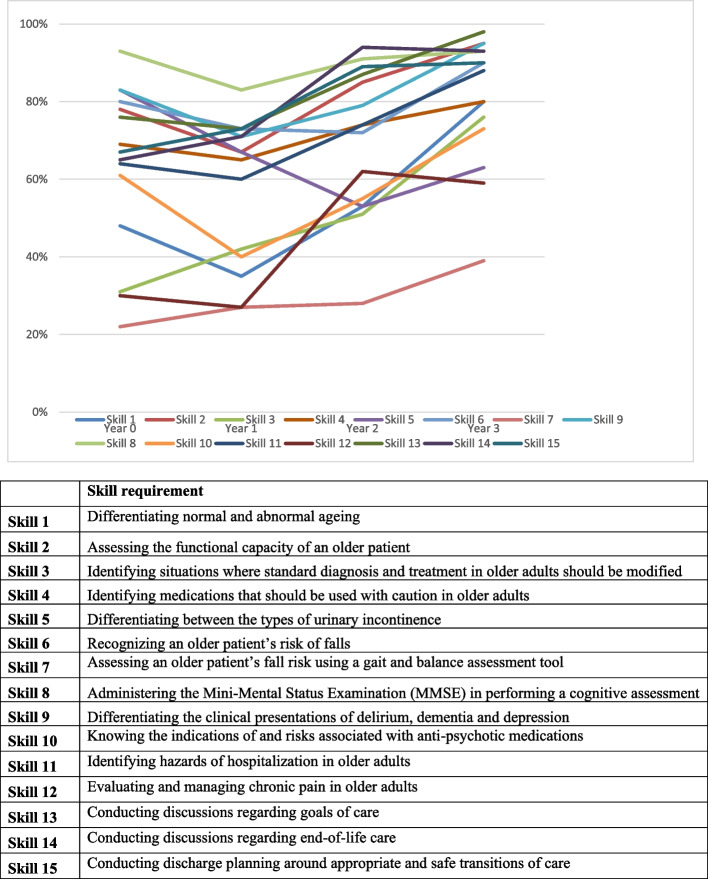
Self-reported Confidence of Internal Medicine Residents (Years 0–3) in Performing Geriatric Skills Using the Geriatric Skills Assessment Tool (GSAT)

This study examined the use of a self-rating instrument, known as the Geriatric Skills Assessment Tool (GSAT) (Fig. 1), among a sample of internal medicine residents in Toronto, Ontario. This tool was developed by Jamshed & Sinha [35] to establish learning needs by assessing self-confidence in performing and teaching geriatric skills and determining interest in acquiring further training in geriatric skills [35, 36].

## Methods

### Study aims and design

This study aimed to describe the self-reported confidence and training needs of internal medicine residents with regard to core geriatric skills, using the GSAT. The specific objectives were to: 1) identify the self-reported confidence of internal medicine residents in performing core geriatric skills; and 2) identify the self-reported geriatric medicine training needs of internal medicine residents. A quantitative study using a non-experimental design was utilized. A novel survey (GSAT) was implemented to answer the research objectives.

### Ethical consideration

This study was approved by the Mount Sinai Hospital Research Ethics Board and was conducted in accordance with all regulations (REB #11–0124-E). All participants provided informed consent by completing the survey.

### Setting

This study explored patterns of confidence and training needs amongst internal medicine residents at the University of Toronto (UT). UT is a world-leading educational institution and the sole medical school for numerous major hospitals in Toronto, Canada’s most populous city. It runs the largest internal medicine residency program in Canada and is in the minority of programs mandating the completion of a rotation in geriatrics, which occurs between years 1 to 3 at a time of the trainees’ choosing [37, 38]. During this rotation, internal medicine residents are required to spend a minimum of one block (~ 4 weeks) in geriatric medicine. This block incorporates providing inpatient and outpatient geriatric medicine consultations and follow-up care, scheduled and informal teaching sessions.

### Participants and data collection

All incoming (Year 0) and current (Years 1–3) core internal medicine residents at UT were invited to complete an anonymous, electronic SurveyMonkey® or paper-based structured survey from February to June 2012. This captured existing internal medicine residents towards the end of their current training year, as well as the newly selected group of incoming internal medicine residents who were final-year medical students at the time of survey distribution. The survey assessed core geriatric skills competencies using the GSAT and collected baseline demographic and training history information.

Survey invitations were delivered via personalized email and an in-person presentation during an internal medicine academic teaching session. Response rates were optimized through five follow-up email invitations. Participants were incentivized in the form of a random draw for gift cards offered to all who submitted a completed survey. Consent was implied if participants proceeded with survey submission.

### Geriatric skills assessment tool (GSAT)

The American Board of Internal Medicine (ABIM) certifies general internists and medical subspecialists who demonstrate the necessary knowledge, skills and attitudes essential for patient care according to their standards of examination. The GSAT is a cross-sectional educational needs assessment tool developed to assess a general internist’s confidence in performing and teaching, as well as their interest in further training, with regard to 15 ABIM-identified core geriatric clinical skills [35, 36]. While no longer publicly accessible through their website, the ABIM previously published 15 minimum geriatric medicine clinical competencies expected by the end of core residency training in internal medicine. These covered fundamental geriatric care domains such as medication management, cognition, comorbidity and end-of-life care. The ABIM clinical competencies also covered core geriatric care domains found in the 26 minimum geriatric competencies established by a working group of educators from internal medicine and family medicine residency programs in 2007 [39]. To the best of our knowledge, to date, these minimum competencies have not changed.

Freely available on the Portal of Geriatrics Online Education, the GSAT is the first self-rating instrument designed to assess geriatric skills in postgraduate internal medicine trainees and specialists. It has been used to assess the skills of internal medicine teaching faculty and residents at academic teaching hospitals in both Canada and the US [36, 40]. The GSAT is a versatile tool that can be easily used to examine several select competencies of interest in an individual program.

The GSAT uses a modified 4-level (low to high) Likert scale [41], through which respondents are asked to self-report their confidence in performing and teaching 15 geriatric skills, as well as their interest in pursuing further training in them [35, 36, 40]. The validity and reliability of the GSAT were previously demonstrated with internal medicine residents and general internists in an academic hospital setting with an established Cronbach’s alpha coefficient of 0.96 [36, 40].

### Statistical analysis

The GSAT data were analyzed with SAS software® using descriptive statistical methodologies to evaluate the trainees’ responses [42]. For the analysis, the 4-level Likert scale responses were grouped into ‘low’ and ‘high’ responses. A score of 1 or 2 on the Likert scale was considered ‘low’ and 3 or 4 considered ‘high’. Mean scores were determined from the grouped scores. Chi-square tests were then used to determine any significant statistical differences in the mean scores between the various demographic factors collected. A *p*-value < 0.05 was considered statistically significant for our analyses [43].

## Results

190 (75.1%) out of the 253 eligible incoming (Year 0) and current internal medicine residents (Years 1–3) completed the survey and are further characterized in Table 1. A minimum and maximum response rate of 67.2 and 81.8% was achieved across the four groups of trainees. 77.4% of the respondents completed medical school in Canada, while the remaining attended international medical schools across 13 different countries. The overall reported interest in becoming specialists in general internal medicine and geriatrics was 17.9 and 2.6% respectively. 34.2% of Year 1 to 3 residents reported having completed a formal training rotation in geriatrics at the time of the survey. The proportion of Year 1, 2 and 3 residents with reported completion of a geriatric rotation was 4.2, 21.3 and 85.4% respectively.

**Table 1:** Characteristics of GSAT Resident Survey Participants (*N* = 190)

Sample Characteristics	Incoming Residents (Year 0) (*N* = 54)	Year 1 Residents (*N* = 48)	Year 2 Residents (*N* = 47)	Year 3 Residents (*N* = 41)
Male, n (%)	39 (72.2)	22 (45.8)	27 (57.4)	21 (51.2)
Canadian medical school training, n (%)	41 (75.9)	37 (77.1)	37 (78.7)	32 (78.0)
Future subspecialty of interest, n (%)				
General internal medicine	7 (13.0)	8 (16.7)	7 (14.9)	12 (29.2)
Geriatric medicine	2 (3.7)	0 (0)	2 (4.3)	1 (2.4)
Undecided	33 (61.1)	18 (37.5)	10 (21.3)	2 (4.9)
Completed geriatric rotation in residency, n (%)	N/A	2 (4.2)	10 (21.3)	35 (85.4)

### Summary of findings around geriatric skills

There was no gender variation other than the observation that female respondents showed a higher interest in acquiring further training about situations where diagnosis and treatment in older adults should be modified (GSAT Skill 3, *P* = 0.03). Additional file 1: Supplemental Table 1 provides the Chi-square results examining differences in the mean scores around the three domains of the 15 geriatric skills observed concerning various demographic factors.

Significant GSAT score differences were found in several selected domains for the respondents’ place of medical school training, prior completion of a geriatric training rotation, number of postgraduate training years, and identified future subspecialty area of interest. Respondents who attended a medical school in Canada were significantly more confident in assessing functional capacity (GSAT Skill 2, *P* = 0.01), administering the Mini-Mental Status Examination (MMSE) [44] (GSAT Skill 8, *P* = 0.004) and conducting discussions regarding goals of care (GSAT Skill 13, *P* = 0.01), compared with respondents who completed medical school outside of Canada. They were also significantly more confident in teaching 5/15 GSAT Skills (*P* < 0.001 to *P* = 0.03).

Year 1–3 respondents who had completed a formal rotation in geriatrics during their residency reported a significantly higher degree of confidence in performing 13/15 GSAT Skills (*P* < 0.001 to *P* = 0.04) and in teaching 9/15 GSAT Skills (*P* < 0.001 to *P* = 0.04), compared to those who had not completed a geriatrics rotation.

The number of postgraduate years in residency training significantly impacted respondents’ confidence in performing and teaching geriatric skills. Incoming residents (Year 0) were more confident in performing 8/15 and teaching 6/15 GSAT Skills when compared with Year 1 residents. Year 2 and Year 3 residents showed higher levels of confidence in performing and teaching 11/15 and 12/15 GSAT Skills respectively compared with lower year residents. There were 4/15 GSAT skills in which Year 1 or 2 residents who had completed a formal rotation in geriatrics showed significantly greater confidence in performing compared to Year 3 residents who had not completed a geriatric rotation. These included identifying medications that should be used with caution in older adults (GSAT Skill 4, *P* = 0.05), assessing an older patient’s fall risk using a gait and balance assessment tool (GSAT Skill 7, *P* = 0.006), knowing the indications of and risks associated with antipsychotic medications (GSAT Skill 10, *P* = 0.02) and evaluating and managing chronic pain in older adults (GSAT Skill 12, *P* < 0.001). Tables 2 and 3 provide mean Likert scores related to confidence in performing and teaching GSAT Skills.

**Table 2:** Mean Likert Scores^a^ Related to Confidence in Performing GSAT Skills

GSAT Skill Item	Year 0 Incoming Residents	Year 1 Residents Mean Confidence in Performing Likert Scale Score	Year 2 Residents Mean Confidence in Performing Likert Scale Score	Year 3 Residents Mean Confidence in Performing Likert Scale Score
Skill 1. Differentiating normal from abnormal aging	2.5	2.3	2.5	2.9
Skill 2. Assessing the functional capacity of an older patient	3.0	2.8	3.0	3.3
Skill 3. Identifying situations where standard diagnosis and treatment in older adults should be modified	2.2	2.3	2.6	2.9
Skill 4. Identifying medications that should be used with caution in older adults	2.8	2.7	2.8	3.0
Skill 5. Differentiating between the types of urinary incontinence	3.0	2.8	2.6	2.7
Skill 6. Recognizing an older patient’s risk of falls	2.9	2.9	2.9	3.3
Skill 7. Assessing an older patient’s fall risk using a gait and balance assessment tool	2.1	2.0	2.1	2.3
Skill 8. Administering the MMSE in performing a cognitive assessment	3.6	3.3	3.4	3.6
Skill 9. Differentiating the clinical presentations of delirium, dementia and depression	3.2	2.8	3.0	3.3
Skill 10. Knowing the indications of and risks associated with anti-psychotic medications	2.7	2.4	2.6	2.8
Skill 11. Anticipating and identifying hazards of hospitalization in older adults	2.8	2.7	2.9	3.2
Skill 12. Evaluating and managing chronic pain in older adults	2.2	2.1	2.7	2.7
Skill 13. Conducting effective discussions regarding goals of care	3.0	2.8	3.2	3.4
Skill 14. Conducting effective discussions regarding end-of-life care	2.7	2.8	3.3	3.3
Skill 15. Conducting good discharge planning around appropriate and safe transitions of care	2.7	2.9	3.1	3.1

^a^4-level Likert scale from low to high. Lower scores indicate lower self-reported confidence

*GSAT* Geriatric Skills Assessment Tool, *MMSE* Mini-Mental Status Examination

**Table 3:** Mean Likert Scores^a^ Related to Confidence in Teaching GSAT Skills

GSAT Skill Item	Year 0 Incoming Residents	Year 1 Residents Mean Confidence in Teaching Likert Scale Score	Year 2 Residents Mean Confidence in Teaching Likert Scale Score	Year 3 Residents Mean Confidence in Teaching Likert Scale Score
Skill 1. Differentiating normal from abnormal aging	2.0	1.6	2.1	2.3
Skill 2. Assessing the functional capacity of an older patient	2.5	2.4	2.8	2.9
Skill 3. Identifying situations where standard diagnosis and treatment in older adults should be modified	1.9	2.0	2.2	2.6
Skill 4. Identifying medications that should be used with caution in older adults	2.4	2.3	2.7	2.9
Skill 5. Differentiating between the types of urinary incontinence	2.7	2.4	2.3	2.4
Skill 6. Recognizing an older patient’s risk of falls	2.5	2.5	2.5	2.8
Skill 7. Assessing an older patient’s fall risk using a gait and balance assessment tool	1.9	2.0	1.9	2.1
Skill 8. Administering the MMSE in performing a cognitive assessment	3.2	3.0	3.1	3.5
Skill 9. Differentiating the clinical presentations of delirium, dementia and depression	2.8	2.6	2.8	3.0
Skill 10. Knowing the indications of and risks associated with anti-psychotic medications	2.3	2.1	2.4	2.6
Skill 11. Anticipating and identifying hazards of hospitalization in older adults	2.4	2.4	2.6	2.9
Skill 12. Evaluating and managing chronic pain in older adults	1.9	2.0	2.3	2.6
Skill 13. Conducting effective discussions regarding goals of care	2.6	2.7	3.1	3.1
Skill 14. Conducting effective discussions regarding end-of-life care	2.5	2.6	3.2	3.2
Skill 15. Conducting good discharge planning around appropriate and safe transitions of care	2.4	2.6	3.0	3.1

^a^4-level Likert scale from low to high. Lower scores indicate lower self-reported confidence

*GSAT* Geriatric Skills Assessment Tool, *MMSE* Mini-Mental Status Examination

Differences were noted in the collective self-rating scores between residents interested in general internal medicine as a future subspecialty versus residents who were reportedly undecided or interested in other subspecialties across all years of training. Those interested in general internal medicine expressed greater confidence in performing 5/15 GSAT Skills (*P* = 0.002 to *P* = 0.05, see Additional file 1: Supplemental Table 1). They were also significantly more confident in teaching skills to identify medications that should be used with caution in older adults (GSAT Skill 4, *P* = 0.02) and recognizing an older person’s risk of falls (GSAT Skill 6, *P* = 0.02). However, these internal medicine residents were less confident in teaching the evaluation and management of chronic pain in older adults when compared with all other residents (GSAT Skill 12, *P* = 0.04). Our results did not show that residents interested in geriatrics expressed a significantly greater interest to pursue further training in core geriatric skills, compared with other residents. Residents who were undecided about their future subspecialty were less confident in differentiating normal from abnormal aging (GSAT Skill 1, *P* = 0.05) and identifying situations where standard diagnosis and treatment in older adults should be modified (GSAT Skill 3, *P* = 0.003).

Table 4 lists the top GSAT skills in which residents reported both their highest and lowest mean scores for their confidence in performing, teaching and interest in further training. Overall, trainees identified their highest confidence in administering the MMSE (GSAT Skill 8, mean Likert score 3.48), performing a goal of care discussion (GSAT Skill 13, mean Likert score 3.09) and differentiating between delirium, dementia and depression (GSAT Skill 9, mean Likert score 3.04). Trainees reported their lowest confidence in assessing fall risk using a gait and balance tool (GSAT Skill 7, mean Likert score 2.13), evaluating and managing chronic pain (GSAT Skill 12, mean Likert score 2.41) and identifying situations where standard diagnosis and treatment should be modified (GSAT Skill 3, mean Likert score 2.46). The GSAT skills in which trainees reported lower confidence in performing were different from the skills in which they reported the highest interest in further training. Trainees reported their highest interest in further training for identifying medications to be used with caution (GSAT Skill 4, mean Likert score 3.41), conducting goals of care discussions (GSAT Skill 13, mean Likert score 3.4) and conducting end-of-life care discussions (GSAT Skill 14, mean Likert score 3.34).

**Table 4:** Top 3 Reported GSAT Skills with Highest and Lowest Overall Mean Scores (*N* = 190)

	Core Geriatric Skill (GSAT Item Number)^a^	Mean Likert Score across all residents
Highest Reported Confidence in Performing	Administering the MMSE (8)	3.48
	Conducting goals of care discussions (13)	3.09
	Differentiating between delirium, dementia and dementia (9)	3.04
Lowest Reported Confidence in Performing	Assessing fall risk using a gait and balance tool (7)	2.13
	Evaluating and managing chronic pain in older adults (12)	2.41
	Identifying situations where standard diagnosis and treatment in older adults should be modified (3)	2.46
Highest Reported Confidence in Teaching	Administering the MMSE (8)	3.18
	Conducting end-of-life discussions (4)	2.84
	Conducting goals of care discussions (13)	2.83
Lowest Reported Confidence in Teaching	Assessing fall risk using a gait and balance tool (7)	1.95
	Differentiating normal and abnormal aging (1)	1.96
	Identifying situations where standard diagnosis and treatments in older adults should be modified (3)	2.1
Highest Reported Interest in Further Training	Identifying medications to be used with caution in the elderly (4)	3.51
	Conducting goals of care discussions (13)	3.4
	Conducting end-of-life discussions (14)	3.34
Lowest Reported Interest in Further Training	Administering the MMSE (8)	2.73
	Differentiating between the types of urinary incontinence (5)	2.82
	Assessing the functional capacity of an older adult (2)	2.89

^a^4-level Likert scale from low to high. Lower scores indicate lower self-reported confidence or interest

*GSAT* Geriatric Skills Assessment Tool, *MMSE* Mini-Mental Status Examination

When examining the responses of Year 3 residents who were in their final year of internal medicine training, their lowest level of confidence was in assessing an older patient’s fall risk using a screening gait and balance assessment tool (GSAT Skill 7, mean Likert score 2.3) and in evaluating and managing chronic pain in older adults (GSAT Skill 12, mean Likert score 2.7). The two GSAT skills with the reported highest interest in acquiring further training were knowing indications and risks associated with antipsychotic medications (GSAT Skill 10, mean Likert score 3.2) and managing chronic pain in older adults (GSAT Skill 12, mean Likert score 3.2) (see Additional file 2: Supplemental Table 2).

## Discussion

Several educational strategies have been devised to improve the ability of internal medicine residents to provide appropriate geriatric care. However, despite numerous endeavors to evaluate the impact of such strategies, no existing study has used the GSAT for this purpose. This study is the largest known survey of incoming and current internal medicine residents about their confidence in performing and teaching, as well as interest in further training, with regard to 15 previously identified ABIM core geriatric skills and competencies. Furthermore, this study’s collection of additional demographic and medical training information enables an understanding of additional factors that may influence the nature of the responses given. The results of this study may be used to inform future research and educational efforts, given that information about the training needs and confidence of internal medicine residents in the field of geriatrics is sparse. This preliminary study highlights perceived gaps in knowledge and skills that may inform professional development approaches for future internal medicine residents and their educators.

Our findings clearly demonstrate that both the overall time spent in postgraduate internal medicine residency training and prior exposure to a formal training rotation in geriatrics significantly improved the self-reported confidence of trainees in performing core geriatric skills. This finding has been confirmed by previous studies, which found the overall time spent in postgraduate residency training improved the self-reported confidence of trainees in surgery [45], family medicine [46] and obstetrics [47]. While growth in confidence appeared to escalate linearly with years of residency training, the incoming (Year 0) trainees declared a slightly higher level of confidence than the Year 1 trainees. This correlates with other medical education studies that document a phenomenon where junior trainees may be unaware of their knowledge gaps and overestimate their abilities [48–51]. Nevertheless, it is clear that as residents progress through their training, they build their confidence in caring for older patients. We note a high degree of association between exposure to geriatric medicine and confidence in caring for older adults; a phenomenon that has largely been studied in the context of undergraduate medical education (e.g., [52]). Most internal medicine residents in this study chose to complete their mandatory geriatric rotation late in residency, with only 21.3% of second-year residents having completed this compared with 85.4% of third-year residents. Of note, completing a formal training rotation in geriatrics had a significant positive impact on trainees’ self-reported confidence around performing 13/15 GSAT skills, compared with trainees who had not yet completed their geriatrics rotation. Additionally, first- and second-year residents who had completed a geriatric rotation demonstrated a greater level of confidence in their knowledge of some geriatric skills compared with third-year residents who had not yet completed this, despite the first-year residents having less overall clinical experience. This suggests that both exposure to geriatric medicine and general clinical experience from accumulating years of residency training increase self-reported confidence in performing geriatric skills. Other authors have similarly noted that an increase in experience and exposure to clinical cases in various specialties (e.g., family medicine, surgery, oncology) can result in increased resident confidence in these areas [31, 53, 54]. Our findings suggest that, in some cases, completing a formal geriatrics rotation may be more crucial in the development of a resident’s geriatric skills than cumulative years of residency experience, supporting the argument for making a geriatrics training curriculum mandatory for all internal medicine residents. Whereby residents identified low confidence, such as in the areas of deprescribing, chronic pain management and fall risk assessment, interprofessional educational experiences with pharmacy and physiotherapy trainees may be an innovative way to meet these training needs [55, 56]. Interprofessional education can also be facilitated through technology, such as having virtual patients [57, 58]. Having interprofessional educators and colleagues has also been found to better prepare residents for collaborative practice in the care of their geriatric patients and increase their confidence about specific geriatric syndromes and practices [59].

Considering that there is still no mandatory national geriatric training requirement for internal medicine residents in Canada [60, 61], and only 43% of internal medicine residents in the United States have previously reported feeling confident in their geriatric skills [62], this study’s findings reinforce the potential benefits of incorporating geriatric training into all internal medicine residency programs. Despite 77% of the residents in our study having graduated from a Canadian medical school, it was clear that they were significantly more confident in performing and teaching core geriatric skills compared to their internationally trained colleagues. Given that geriatrics is a relatively new specialty, some countries do not incorporate geriatrics into their medical school curriculum, which may account for this finding [63]. Our findings may reflect the concerted efforts undertaken in Canada [54] to identify core-training competencies for medical students and encourage the implementation of training opportunities in medical schools to support the better provision of geriatric care [55, 64–67]. This study highlights not only the importance of dedicated training to build residents’ confidence in essential geriatric skills, but suggests that exposing trainees to formal geriatrics training early in their residencies may help them to more quickly master these skills.

When examining respondents’ future subspecialty areas of interest, those who expressed an interest in becoming a general internist were more likely to report greater confidence in performing and teaching most geriatric skills, compared to those who were undecided. While it may be expected that those declaring a future interest in geriatrics should have had the greatest self-reported confidence, there was insufficient statistical power to confirm or refute this expectation as only 2.6% of the respondents fell into this category. As a result, future studies are encouraged to explore the association between self-reported confidence in geriatrics and career-practice preferences during internal medicine residencies.

By evaluating self-reported confidence with and interest in learning about various geriatric skills, this study shows how the GSAT can be used to inform the design of a geriatric medicine training curriculum that is tailored to the learning needs of internal medicine residents. For example, respondents in our study had the highest reported confidence in both performing and teaching, as well as their lowest interest in further training on, how to administer the MMSE (GSAT Skill 8), suggesting that further education in this area is not required. Additionally, a topic that third-year residents expressed great interest in receiving further training in, was evaluating and managing chronic pain in older adults (GSAT Skill 12). This was the same geriatric skill in which first and second-year residents who had completed a rotation in geriatrics were significantly more confident in managing than third-year residents who had not completed a geriatrics rotation. This type of information may prompt educators to incorporate training on chronic pain earlier in residency to more proactively and adequately address this training gap. Identifying residents’ learning interests and knowledge gaps, as evidenced by low self-reported confidence levels, can also be used to make improvements to internal medicine residency program curricula.

### Study limitations

As a cross-sectional, single-site study providing a descriptive snapshot of UT internal medicine residents, our findings have training- and location-specific implications that may not be transferable to other programs that vary in size, geographical location, curriculum orientation, and culture. However, it is important to note that our survey achieved a 75% overall response rate and was conducted in one of the largest internal medicine resident cohorts in North America, with trainees who had completed undergraduate training in over 38 medical schools across 14 countries around the world. Further research is needed to develop a broader understanding of the geriatric training needs and applicability of the GSAT in determining these amongst internal medicine residents in other settings. A further limitation is that the GSAT is currently not a validated tool, despite being used in previous research [36], and although correlations were identified between the variables we assessed, the study and survey itself does not allow us to establish causal relationships. It is also important to note that we have not established any connection between the perceived confidence level and the actual geriatric medicine skill level of the residents we surveyed. Finally, data collected from this study is now several years old. It is likely that internal medicine residency programs, including that at UT, have changed over this time. The results of and conclusions drawn from this study may have had a greater impact, particularly on local practice, had they been published in closer temporal proximity to when it was conducted. Nonetheless, highlighting the important role a needs assessment can potentially have to improve residency training programs, and demonstrating how the GSAT can be used to achieve this for geriatric medicine, still remains highly relevant to this day.

## Conclusion

This study emphasizes the value of a needs assessment, such as the GSAT, in the improvement of postgraduate medical curricula. Using the GSAT amongst all UT internal medicine residents, our findings suggest that the inclusion of a formal rotation in geriatrics, particularly early on in training, significantly improves residents’ confidence in performing and teaching multiple core geriatric skills. Medical curricula designers should consider incorporating a mandatory geriatric rotation for all internal medicine residents. Future research is encouraged to consider the applicability of the GSAT in determining training needs amongst internal medicine residents in other settings, such that this information can be used to further refine recommendations for internal medicine training.

## Supplementary Information

**Additional file 1.**

**Additional file 2.**

**Additional file 3.**

## Data Availability

The datasets used and/or analyzed during the current study are available from the corresponding author on reasonable request.

## Abbreviations

GSAT: Geriatric Skills Assessment Tool
MMSE: Mini-Mental State Exam
