# Supplementary material for: Identifying the areas of low self-reported confidence of internal medicine residents in geriatrics: a descriptive study of findings from a structured geriatrics skills assessment survey
Source: BMC Med Educ. 2022 Dec 15;22:870. doi: 10.1186/s12909-022-03934-2 (PMC9756669; doi:10.1186/s12909-022-03934-2)
Supplement: Supplementary file 1 — Additional file 1. [file 12909_2022_3934_MOESM1_ESM.docx]

| Additional file 1. Chi-square P Value Results Between GSAT Skill Items and Variables | | | | | | |
| --- | --- | --- | --- | --- | --- | --- |
| Skill Item | Canadian Medical School Graduates with High Confidence or Interest in GSAT Skill | Residents with Completed Geriatric Rotation with High Confidence or Interest in GSAT Skill | Completed Geriatric Rotation Year 1 or 2 Residents with High Confidence or Interest in GSAT Skill Compared with Year 3 Residents with No Geriatric Rotation | Year of Residency Training | Residents Interested in General Internal Medicine with High Confidence or Interest in GSAT Skill | Residents Interested in Geriatric Medicine with High Confidence or Interest in GSAT Skill |
| Skill 1. Differentiating normal from abnormal aging. | | | | | | |
| Confidence performing skill | 0.96 | < 0.001 | 0.55 | < 0.001^a^ | 0.06 | 0.22 |
| Interest in further training | 0.28 | 0.20 | 0.30 | 0.15 | 0.06 | 0.30 |
| Confidence teaching skill | 0.63 | 0.04 | 0.47 | 0.03^a^ | 0.14 | 0.03 |
| Skill 2. Assessing the functional capacity of an older patient. | | | | | | |
| Confidence performing skill | 0.01 | < 0.001 | 0.18 | 0.007^a^ | 0.03 | 0.26 |
| Interest in further training | 0.17 | 0.13 | 0.02 | 0.005^a^ | 0.62 | 0.62 |
| Confidence in teaching skill | <0.001 | 0.28 | 0.26 | 0.004^a^ | 0.65 | 0.28 |
| Skill 3. Identifying situations where standard diagnosis and treatment in older adults should be modified. | | | | | | |
| Confidence performing skill | 0.27 | < 0.001 | 0.15 | < 0.001^a^ | 0.56 | 0.15 |
| Interest in further training | 0.60 | 0.95 | 0.29 | 0.27 | 0.16 | 0.31 |
| Confidence teaching skill | 0.37 | < 0.001 | 0.06 | < 0.001^a^ | 0.37 | 0.15 |
| Skill 4. Identifying medications that should be used with caution in older adults. | | | | | | |
| Confidence performing skill | 0.45 | 0.01 | 0.05 | 0.39 | 0.006 | 0.16 |
| Interest in further training | 0.09 | 0.52 | 0.06 | 0.18 | 0.48 | 0.47 |
| Confidence teaching skill | 0.82 | < 0.001 | 0.63 | < 0.001^a^ | 0.02 | 0.22 |
| Skill 5. Differentiating between the types of urinary incontinence. | | | | | | |
| Confidence performing skill | 0.72 | 0.69 | 0.30 | 0.01^a^ | 0.10 | 0.54 |
| Interest in further training | 0.16 | 0.31 | 0.42 | 0.02^a^ | 0.24 | 0.56 |
| Confidence teaching skill | 0.72 | 0.80 | 0.79 | 0.21 | 0.61 | 0.69 |
| Skill 6. Recognizing an older patient’s risk of falls. | | | | | | |
| Confidence performing skill | 0.76 | < 0.001 | 0.25 | 0.15 | 0.12 | 0.93 |
| Interest in further training | 0.61 | 0.51 | 0.25 | 0.06 | 0.45 | 0.27 |
| Confidence teaching skill | 0.09 | 0.01 | 0.21 | 0.27 | 0.02 | 0.22 |
| Skill 7. Assessing an older patient’s fall risk using a gait and balance assessment tool. | | | | | | |
| Confidence performing skill | 0.10 | 0.005 | 0.006 | 0.37 | 0.18 | 0.12 |
| Interest in further training | 0.50 | 0.59 | 0.18 | 0.70 | 0.04 | 0.22 |
| Confidence teaching skill | 0.31 | 0.06 | 0.33 | 0.31 | 0.80 | 0.32 |
| Skill 8. Administering the MMSE in performing a cognitive assessment. | | | | | | |
| Confidence performing skill | 0.004 | 0.03 | 0.18 | 0.26 | 0.42 | 0.46 |
| Interest in further training | 0.54 | 0.19 | 0.52 | 0.02^a^ | 0.99 | 0.33 |
| Confidence teaching skill | < 0.001 | 0.10 | 0.42 | 0.03^a^ | 0.38 | 0.31 |
| Skill 9. Differentiating the clinical presentations of delirium, dementia and depression. | | | | | | |
| Confidence performing skill | 0.07 | 0.006 | 0.70 | 0.03^a^ | 0.002 | 0.28 |
| Interest in further training | 0.21 | 0.61 | 0.25 | 0.31 | 0.14 | 0.33 |
| Confidence teaching skill | 0.03 | 0.01 | 0.55 | 0.04^a^ | 0.13 | 0.67 |
| Skill 10. Knowing the indications of and risks associated with anti-psychotic medications. | | | | | | |
| Confidence performing skill | 0.37 | < 0.001 | 0.02 | 0.01^a^ | 0.31 | 0.29 |
| Interest in further training | 0.60 | 0.23 | 0.70 | 0.76 | 0.53 | 0.48 |
| Confidence teaching skill | 0.74 | 0.01 | 0.52 | 0.009^a^ | 0.33 | 0.99 |
| Skill 11. Anticipating and identifying hazards of hospitalization in older adults. | | | | | | |
| Confidence performing skill | 0.28 | 0.003 | 0.25 | 0.02^a^ | 0.71 | 0.15 |
| Interest in further training | 0.96 | 0.89 | 0.24 | 0.29 | 0.42 | 0.83 |
| Confidence teaching skill | 0.51 | 0.04 | 0.70 | 0.004^a^ | 0.34 | 0.25 |
| Skill 12. Evaluating and managing chronic pain in older adults. | | | | | | |
| Confidence performing skill | 0.39 | < 0.001 | < 0.001 | < 0.001^a^ | 0.52 | 0.44 |
| Interest in further training | 0.45 | 0.65 | 0.70 | 0.93 | 0.53 | 0.34 |
| Confidence teaching skill | 0.71 | < 0001 | 0.30 | < 0.001^a^ | 0.04^b^ | 0.19 |
| Skill 13. Conducting effective discussions regarding goals of care. | | | | | | |
| Confidence performing skill | 0.01 | 0.003 | 0.18 | 0.008^a^ | 0.05 | 0.30 |
| Interest in further training | 0.20 | 0.41 | 0.55 | 0.03^a^ | 0.33 | 0.62 |
| Confidence teaching skill | < 0.001 | 0.29 | 0.18 | 0.01^a^ | 0.40 | 0.14 |
| Skill 14. Conducting effective discussions regarding end-of-life care. | | | | | | |
| Confidence performing skill | 0.07 | 0.04 | 0.18 | <0.001^a^ | 0.16 | 0.25 |
| Interest in further training | 0.45 | 0.07 | 0.89 | 0.02^a^ | 0.31 | 0.45 |
| Confidence teaching skill | 0.12 | 0.03 | 0.051 | <0.001^a^ | 0.18 | 0.14 |
| Skill 15. Conducting good discharge planning around appropriate and safe transitions of care. | | | | | | |
| Confidence performing skill | 0.21 | 0.08 | 0.051 | 0.007^a^ | 0.02 | 0.24 |
| Interest in further training | 0.66 | 0.23 | 0.87 | 0.40 | 0.53 | 0.71 |
| Confidence teaching skill | 0.02 | 0.10 | 0.55 | <0.001^a^ | 0.54 | 0.10 |

^a^Significant difference in confidence or interest in GSAT skill between the residency years.

^b^Residents interested in general internal medicine were significantly less confident in teaching the evaluation and management of chronic pain compared with other residents (GSAT skill 12).

GSAT = Geriatric Skills Assessment Tool; MMSE = Mini-Mental Status Examination
