# Supplementary material for: Identifying the areas of low self-reported confidence of internal medicine residents in geriatrics: a descriptive study of findings from a structured geriatrics skills assessment survey
Source: BMC Med Educ. 2022 Dec 15;22:870. doi: 10.1186/s12909-022-03934-2 (PMC9756669; doi:10.1186/s12909-022-03934-2)
Supplement: Supplementary file 2 — Additional file 2. [file 12909_2022_3934_MOESM2_ESM.docx]

| Supplemental Table 2. Mean Likert Scores^a^ Related to Interest in Further Training of GSAT Skills | | | | |
| --- | --- | --- | --- | --- |
| GSAT Skill Item | Year 0 Incoming Residents | Year 1 Residents Mean Interest in Further Training Likert Scale Score | Year 2 Residents Mean Interest in Further Training Likert Scale Score | Year 3 Residents Mean Interest in Further Training Likert Scale Score |
| Skill 1. Differentiating normal from abnormal aging | 3.2 | 2.9 | 3.2 | 2.9 |
| Skill 2. Assessing the functional capacity of an older patient | 3.1 | 2.7 | 3.1 | 2.6 |
| Skill 3. Identifying situations where standard diagnosis and treatment in older adults should be modified | 3.4 | 3.0 | 3.2 | 3.1 |
| Skill 4. Identifying medications that should be used with caution in older adults | 3.5 | 3.5 | 3.7 | 2.3 |
| Skill 5. Differentiating between the types of urinary incontinence | 3.0 | 2.8 | 2.9 | 2.5 |
| Skill 6. Recognizing an older patient’s risk of falls | 3.3 | 3.0 | 3.3 | 2.9 |
| Skill 7. Assessing an older patient’s fall risk using a gait and balance assessment tool | 3.1 | 3.0 | 3.2 | 2.9 |
| Skill 8. Administering the MMSE in performing a cognitive assessment | 3.1 | 2.8 | 2.6 | 2.3 |
| Skill 9. Differentiating the clinical presentations of delirium, dementia and depression | 3.4 | 3.3 | 3.3 | 3.0 |
| Skill 10. Knowing the indications of and risks associated with anti-psychotic medications | 3.5 | 3.3 | 3.4 | 3.2 |
| Skill 11. Anticipating and identifying hazards of hospitalization in older adults | 3.4 | 3.0 | 3.1 | 3.1 |
| Skill 12. Evaluating and managing chronic pain in older adults | 3.3 | 3.3 | 3.2 | 3.2 |
| Skill 13. Conducting effective discussions regarding goals of care | 3.6 | 3.4 | 3.2 | 3.1 |
| Skill 14. Conducting effective discussions regarding end-of-life care | 3.6 | 3.5 | 3.3 | 3.1 |
| Skill 15. Conducting good discharge planning around appropriate and safe transitions of care | 3.4 | 3.3 | 3.3 | 3.1 |

^a^4-level Likert scale from low to high. Lower scores indicate lower self-reported interest.

GSAT = Geriatric Skills Assessment Tool; MMSE = Mini-Mental Status Examination
